# Effects in cigarette smoke stimulated bronchial epithelial cells of a corticosteroid entrapped into nanostructured lipid carriers

**DOI:** 10.1186/s12951-014-0046-4

**Published:** 2014-11-29

**Authors:** Maria Luisa Bondì, Maria Ferraro, Serena Di Vincenzo, Stefania Gerbino, Gennara Cavallaro, Gaetano Giammona, Chiara Botto, Mark Gjomarkaj, Elisabetta Pace

**Affiliations:** Istituto per lo Studio dei Materiali Nanostrutturati- U.O.S. di Palermo-Consiglio Nazionale delle Ricerche-via Ugo La Malfa, 153 90146 Palermo, Italy; Istituto di Biomedicina e Immunologia Molecolare-Consiglio Nazionale delle Ricerche – via Ugo La Malfa, 153 90146 Palermo, Italy; Laboratory of Biocompatible Polymers-Dipartimento di Scienze e Tecnologie, Biologiche, Chimiche e Farmaceutiche (STEBICEF), Università di Palermo -via Archirafi, 32-90123 Palermo, Italy

**Keywords:** Nanostructured lipid carriers, Corticosteroid, Fluticasone propionate, Cigarette smoke, Airway epithelial cell, Chronic obstructive pulmonary disease, Asthma

## Abstract

**Background:**

Nanomedicine studies have showed a great potential for drug delivery into the lung. In this manuscript nanostructured lipid carriers (NLC) containing Fluticasone propionate (FP) were prepared and their biocompatibility and effects in a human bronchial epithelial cell line (16-HBE) stimulated with cigarette smoke extracts (CSE) were tested.

**Results:**

Biocompatibility studies showed that the NLC did not induce cell necrosis or apoptosis. Moreover, it was confirmed that CSE increased intracellular ROS production and TLR4 expression in bronchial epithelial cells and that FP-loaded NLC were more effective than free drug in modulating these processes. Finally, the nanoparticles increased GSH levels improving cell protection against oxidative stress.

**Conclusions:**

The present study shows that NLC may be considered a promising strategy to improve corticosteroid mediated effects in cellular models associated to corticosteroid resistance. The NLC containing FP can be considered good systems for dosage forms useful for increasing the effectiveness of fluticasone decreasing its side effects.

## Background

Pulmonary drug delivery is an important research area with a potential high impact in the treatment of various obstructive pulmonary diseases including asthma and chronic obstructive pulmonary disease. It can provide rapid responses and can minimize the required drug dose being the drug delivered directly into the lungs and specifically at the site of activity [1].

Nanomedicine is used for modified and targeted drug delivery. It is based on nanostructured materials at colloidal size (1–500 nm) and is able to release biologically active agents, chemically or physically incorporated, into specific sites and within very well defined time frames. These systems are characterized by: 1) nanoscaled dimensions, able to allow their direct interaction at molecular levels with cell components of the damaged tissue; 2) the ability to incorporate elevated amounts of active molecules with subsequent increase of the efficiency of the drug delivery systems; 3) the ability to deliver the drugs by increasing their bioavailability and decreasing administered doses; 4) the ability to obtain an efficient localization of the drug in the target site [2]. Nanomedicine provides new solutions to clinical problems, particularly in pulmonary diseases, promising better delivery of therapeutics to disease sites [3,4]. These advantages can be properly exploited for the administration of inhaled corticosteroids, especially during long-term therapies like in patients with chronic obstructive pulmonary disease (COPD); potentially, it might be possible to utilise these nanosystems in inhalatory therapies in order to maximize local effects into the lung and to reduce systemic effects as well as the frequency of administration. Moreover, long-term use of high-dose inhaled corticosteroids (ICS) has the potential to cause undesirable side effects. Conversely, a modified delivery system provides constant levels of drug at the prime site of action for a prolonged time and it would enable better control of the disease [5,6].

To obtain these results it is important the choice of the material forming the nanodevices. In particular the use of pegylated lipid for the production of Nanostructured Lipid Carrier (NLC) combines the advantages of the safety of lipids and the possibility of large-scale production, with the mucoadhesive properties useful for improving residence time of nanodevices on airways surface and to contrast the effect of the abnormal production of mucus, occurring in COPD, with the consequent dramatic reduction of corticosteroids absorption [7]. In this context, colloidal lipid nanoparticles such as NLC could give great benefit in designing new drug delivery systems with great potential advantages.

The aim of the present work was to realize a novel drug delivery system to improve the drug bioavailability, making the drug able to achieve an increase of permeability through the membrane cell and consequently to reduce the administered dose. In this paper we report the preparation and characterization of NLC by using a pegylated lipid such as Compritol HD5 ATO, for the delivery through inhalator route of Fluticasone propionate (FP). Pegylated NLC containing Fluticasone (FP-loaded NLC) as well as empty NLC, as control, were prepared and characterized in terms of size, polydispersity index (PDI), surface charge, stability, and in vitro drug release. Moreover, the biological efficiency of this new drug delivery system was evaluated in vitro by using the human bronchial epithelial cell line (16-HBE) considering the effects of the drug, in the loaded-NLC or free form on Reactive Oxygen Species (ROS) production, GSH levels, and TRL4 expression in cigarette smoke extracts (CSE) stimulated cells.

## Results and discussion

In this paper, in order to improve the FP efficacy in the treatment of respiratory diseases such as COPD, FP-loaded NLC were developed. Due to the lypophilic characteristics of FP, FP-loaded NLC were prepared by the precipitation method [8-10]. In particular, Compritol HD5 ATO was chosen as lipid matrix for obtaining NLC with or without FP because of its good biocompatibility and the presence of PEG in its structure. In this regards, it has been shown that the PEG chains can play an important role in transport across mucosae of nanoparticles since their presence can improve their transport across the nasal epithelia [7,11-13].

Since some physicochemical and technological properties are quite critical for biopharmaceutical behaviour of NLC, either empty and drug-loaded samples, after preparation and purification, were characterized in terms of particle size, PDI and ζ potential in three different dispersing aqueous media by light scattering measurements, and analytical data are reported in Table 1.

**Table 1:** **Mean size (nm), polydispersity index (PDI) and ζ-potential (mV) in bi-distilled water, phosphate buffer solution (PBS) and NaCl 0.9 wt% of empty and Fluticasone propionate (FP)-loaded nanoparticles**

**Sample**	**Dispersing medium**	**Mean size (nm)**	**PDI**	**Zeta potential (mV)(± S.D.)**	***EE%*** **(w/w)**
**Empty**	PBS pH 7.4	133.7	0.243	−15.1 ± 3.78	-----
	NaCl 0.9%	132.5	0.215	−12.3 ± 4.16	-----
	H_2_O	115.9	0.285	−27.8 ± 3.21	-----
**FP-loaded**	PBS pH 7.4	178.7	0.266	−14.3 ± 2.44	76.8 ± 0.04
	NaCl 0.9%	189.6	0.244	−13.3 ± 4.56	76.8 ± 0.05
	H_2_O	129.9	0.330	−31.3 ± 4.50	76.8 ± 0.04
**Empty 4 months)**	H_2_O	137.9	0.247	−26.6 ± 3.13	-----
**Empty (10 months)**	H_2_O	149.9	0.235	−25.4 ± 2.15	-----
**FP-loaded (4 months)**	H_2_O	143.2	0.323	−28.3 ± 2.72	74.7 ± 0.06
**FP-loaded (10 months)**	H_2_O	145.6	0.334	−27.5 ± 4.50	72.5 ± 0.03

Efficiency entrapment % *(EE%)* of FP-loaded NLC.

Empty and drug loaded-NLC have size of about 116 and 130 nm in bi-distilled water respectively, and greater in the all other investigated media; these differences could be attributed to the different ionic strength of the media. Moreover, all these systems possessed quite low PDI values, which indicated a good dimensional homogeneity of particles that, together with small size, make them suitable for inhalatory administration.

The ζ potential values of these structures, also reported in Table 1, were rather high (absolute value) in bi-distilled water and decreased when they were determined in PBS and NaCl 0.9% aqueous solution. The presence of electrolytes causes a diminution of surface charge for the potential screening effect of solution ions. The surface charge of nanoparticles is important because it makes the nanosystems more stable when dispersed into an aqueous solution, reducing the occurrence of the aggregation phenomenon. Several systems were prepared with different size and surface characteristics (data not shown) but for the in vitro tests the system with better physical-chemical characteristics was chosen.

**Figure 1:**
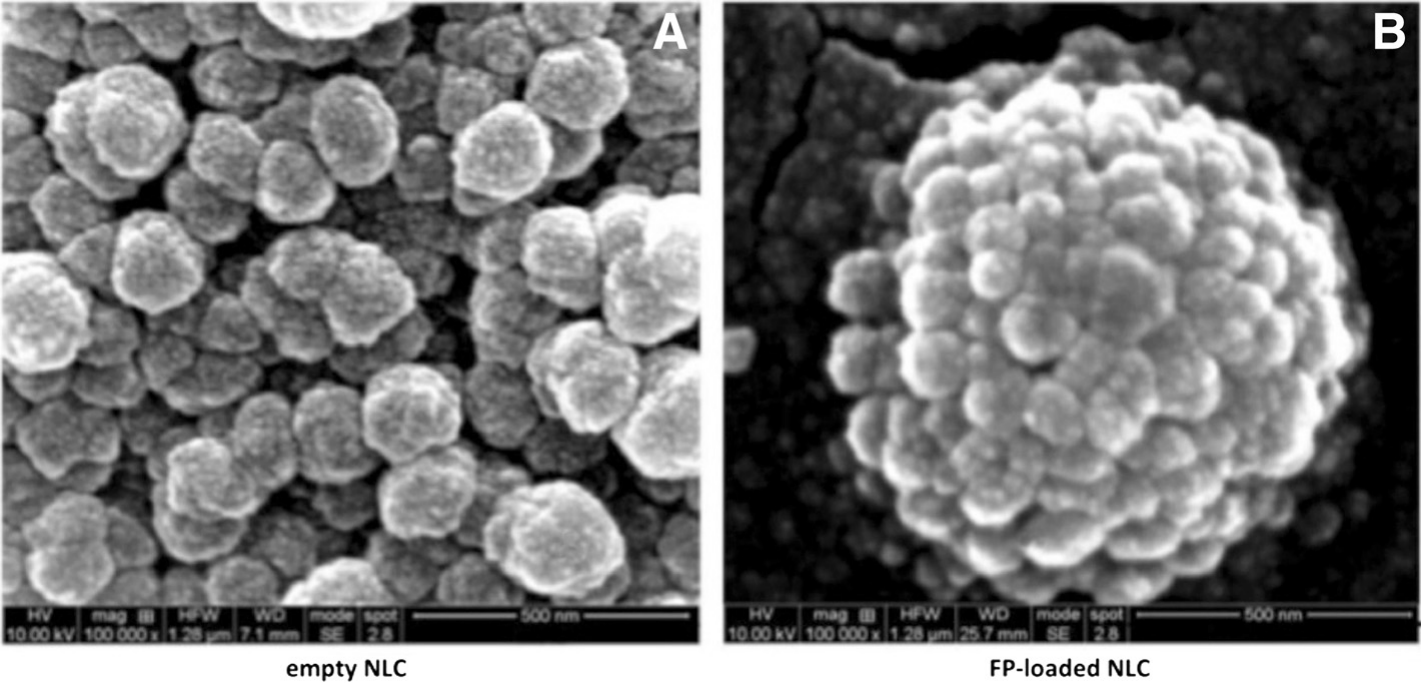
**Scanning electron microscopy.** Representative SEM images of NLC, empty **(A)** and loaded with FP **(B)**, respectively. The bars represent 500 nm.

In order to confirm the nanometer size and to investigate the morphology of empty or FP-loaded NLC, SEM was used and the obtained images are reported in Figure 1.

These images were consistent with the findings obtained from dimensional analysis and also revealed a spherical shape of investigated samples.

Moreover, an important aspect to be taken into account in the formulations of NLC as possible carrier to be aerosolized for the pulmonary delivery of drugs is their capability to give colloidal dispersions stable during storage. The occurrence of aggregation phenomena can lead to a significant worsening of the biopharmaceutical features of colloidal suspensions, above all in terms of ability to be uptaken into the cells.

Therefore in order to evaluate the stability of these systems during storage empty and FP-loaded NLC were kept for 4 months and 10 months at 4°C and subsequently characterized in terms of size, PDI, and ζ potential. The results (Table 1) showed that either empty or FP-loaded NLC were stable during storage under tested conditions.

**Figure 2:**
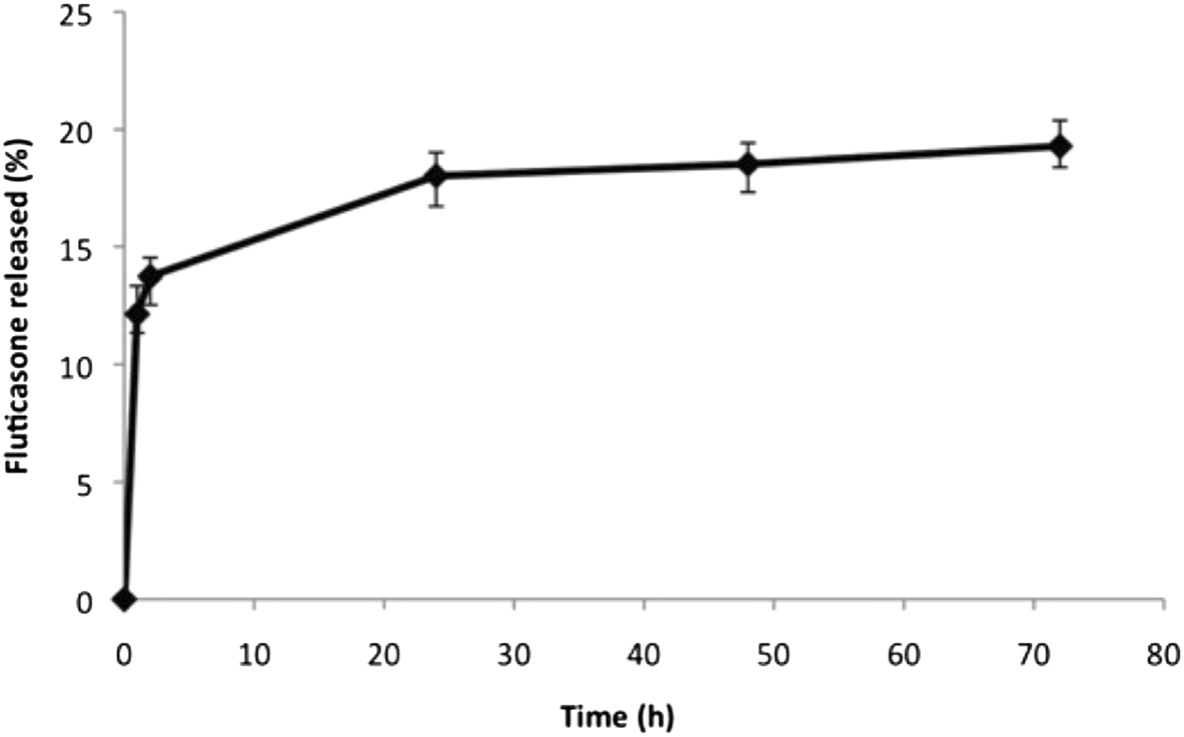
**Drug release profile from FP- loaded NLC.**

*DL %* and *EE %* of FP loaded in NLC were equal to about as 4.8% and 76.8% respectively. In order to evaluate the ability of these NLC of retaining the encapsulated drug under sink conditions and to release it slowly in physiological media, a release study was carried out in PBS at pH 7.4/ethanol mixture 80:20 (v/v) by evaluating the amount of released drug from NLC at prefixed time intervals across a dialysis tube (Spectra/Por®, MWCO 12,000-14,000 Da), in accordance to the European Pharmacopoeia [14,15]. In Figure 2, the drug release profile from FP-loaded NLC was reported until 72 hrs incubation.

As shown, after 1 hr, the amount of FP released from NLC was equal to 15%. An initial burst effect in the drug-release profile of FP-NLC is evident and it can be probably ascribed to the presence of the drug absorbed on the nanoparticle surface. Moreover, these studies revealed that in a physiological-mimicking medium, FP was not completely released from nanoparticles until 72 hrs, supporting the hypothesis that these systems at the contact with the airways mucosae, could efficiently enter in the colloidal form improving then the drug internalization into the cells and its accumulation into human bronchial epithelial cells. The drug burst release shown in this study could be exploited to deliver a high initial dose when desired. The gradual release after the initial burst would also be important in order to maintain an effective drug concentration in the target organ.

An innovative drug delivery system has to be tested for its safety. This aspect is much more relevant in the case of pulmonary delivery, since several side effects may result from an unsafe material [16]. Taking also into account the possibility to incorporate into aerosol droplets the FP-loaded pegylated nanoparticles and to administer them by inhalation, safety of empty NLC and FP-loaded NLC was evaluated *in vitro* by using 16-HBE cells as a model of epithelial cells.

**Figure 3:**
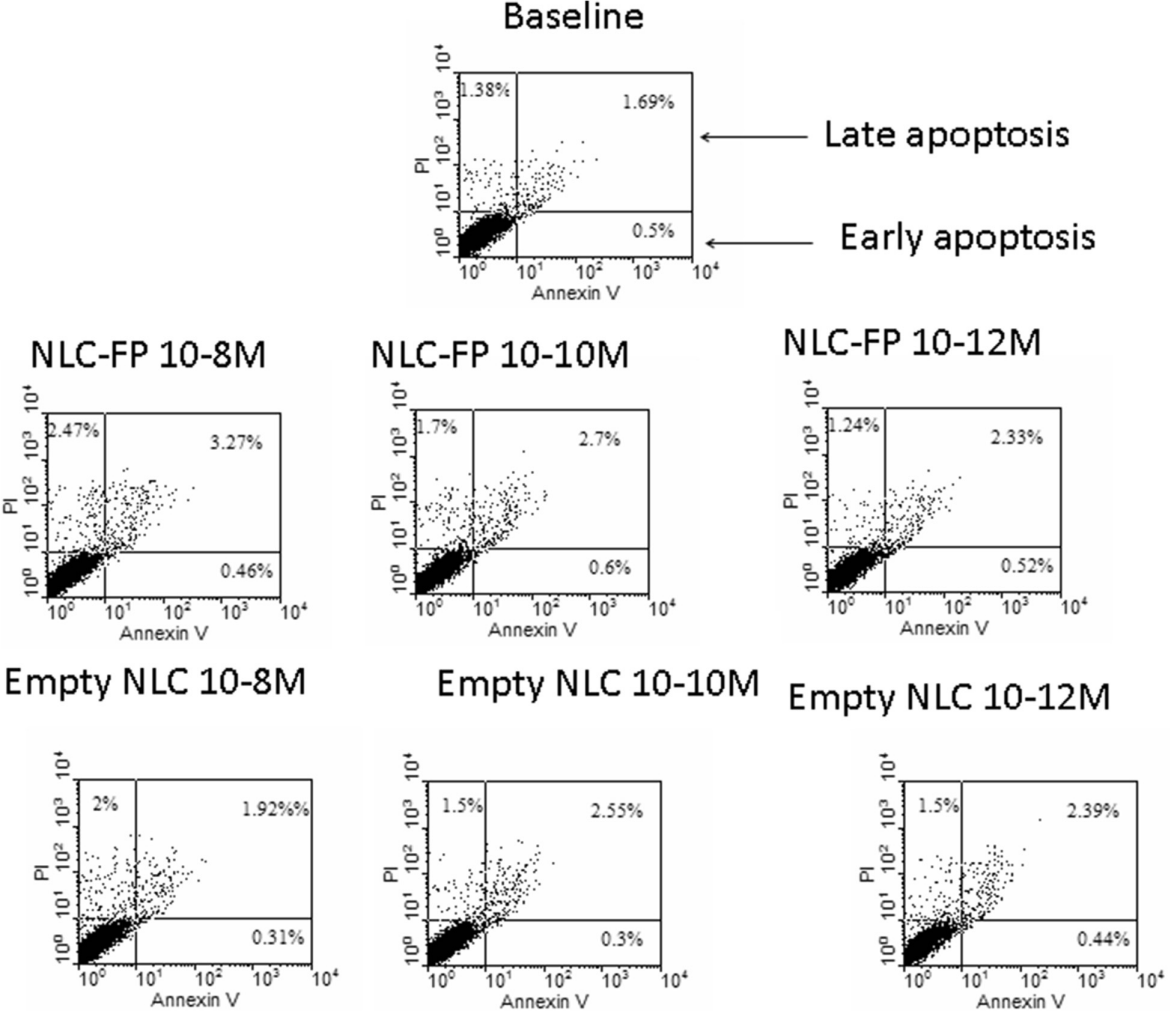
**Biocompatibility of empty and FP- loaded NLC: dose–response experiments.** Bronchial epithelial cells (16-HBE) were cultured in the presence and in the absence of NLC and FP-loaded NLC (10^−8^M, 10^−10^M, 10^−12^M) for 24 hours and cell necrosis and cell apotosis were assessed using the PI/Annexin V method by flow cytometry. Representative dot plots were shown.

**Figure 4:**
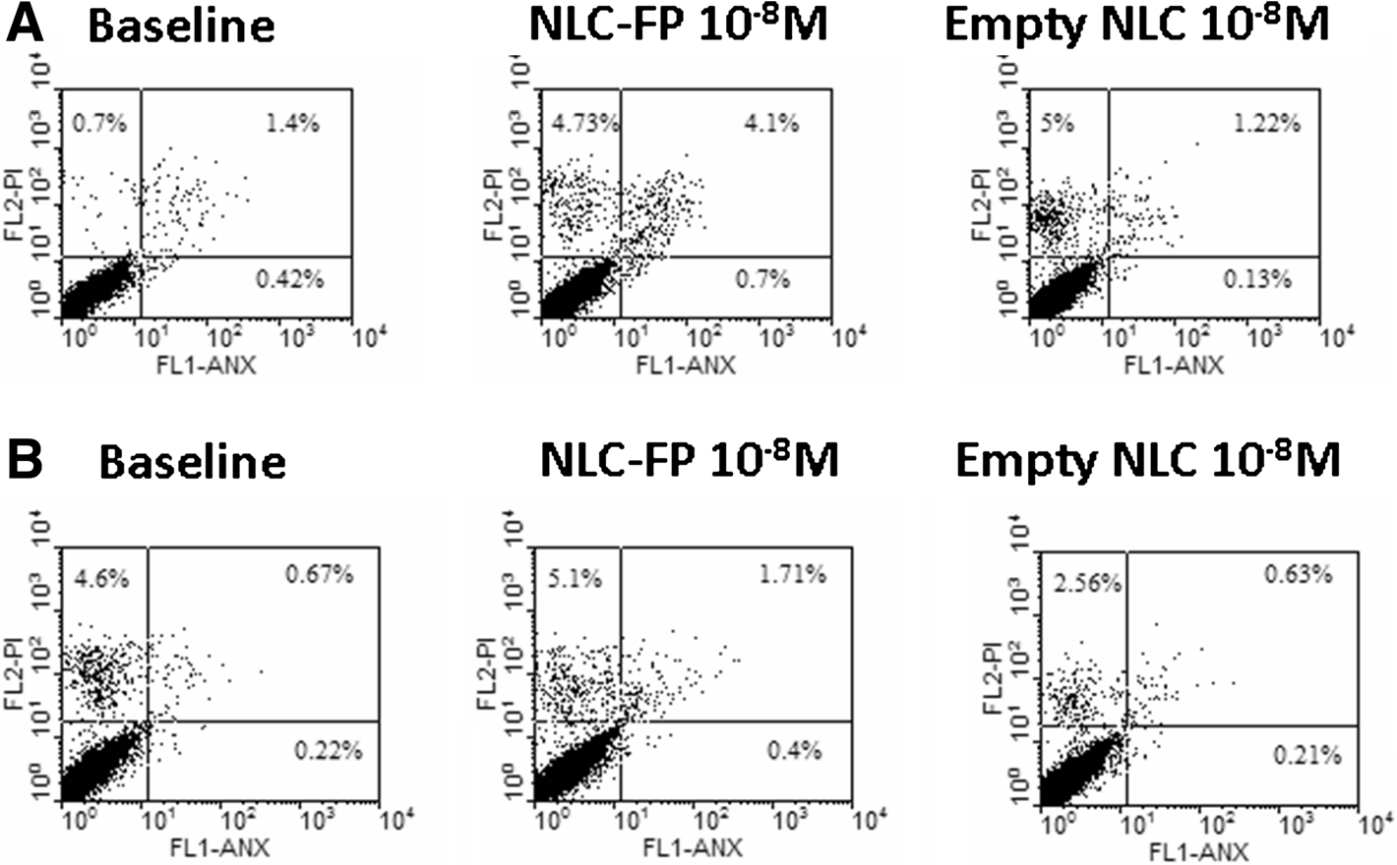
**Biocompatibility of empty and FP- loaded NLC: time-dependent experiments.** Bronchial epithelial cells (16-HBE) were cultured in the presence and in the absence of FP, NLC and FP-loaded NLC (10^−8^M) for 48 **(A)** and 72 **(B)** hours and cell necrosis and cell apotosis were assessed using the PI/Annexin V method by flow cytometry. Representative dot plots were shown.

Cytotoxicity of FP-loaded pegylated nanoparticles in 16-HBE cells (Figures 3 and 4) was evaluated by using the PI/Annexin V binding method [17].

Neither FP-loaded NLC nor empty NLC at both tested concentrations and time points induced relevant numbers of necrotic (PI positive) or of apoptotic (Annexin V positive) 16-HBE cells (Figures 3 and 4), evidencing the high biocompatibility of obtained nanoparticles.

Our results indicate the potential of the obtained NLC as carriers for FP delivered by intra-bronchial route.

**Figure 5:**
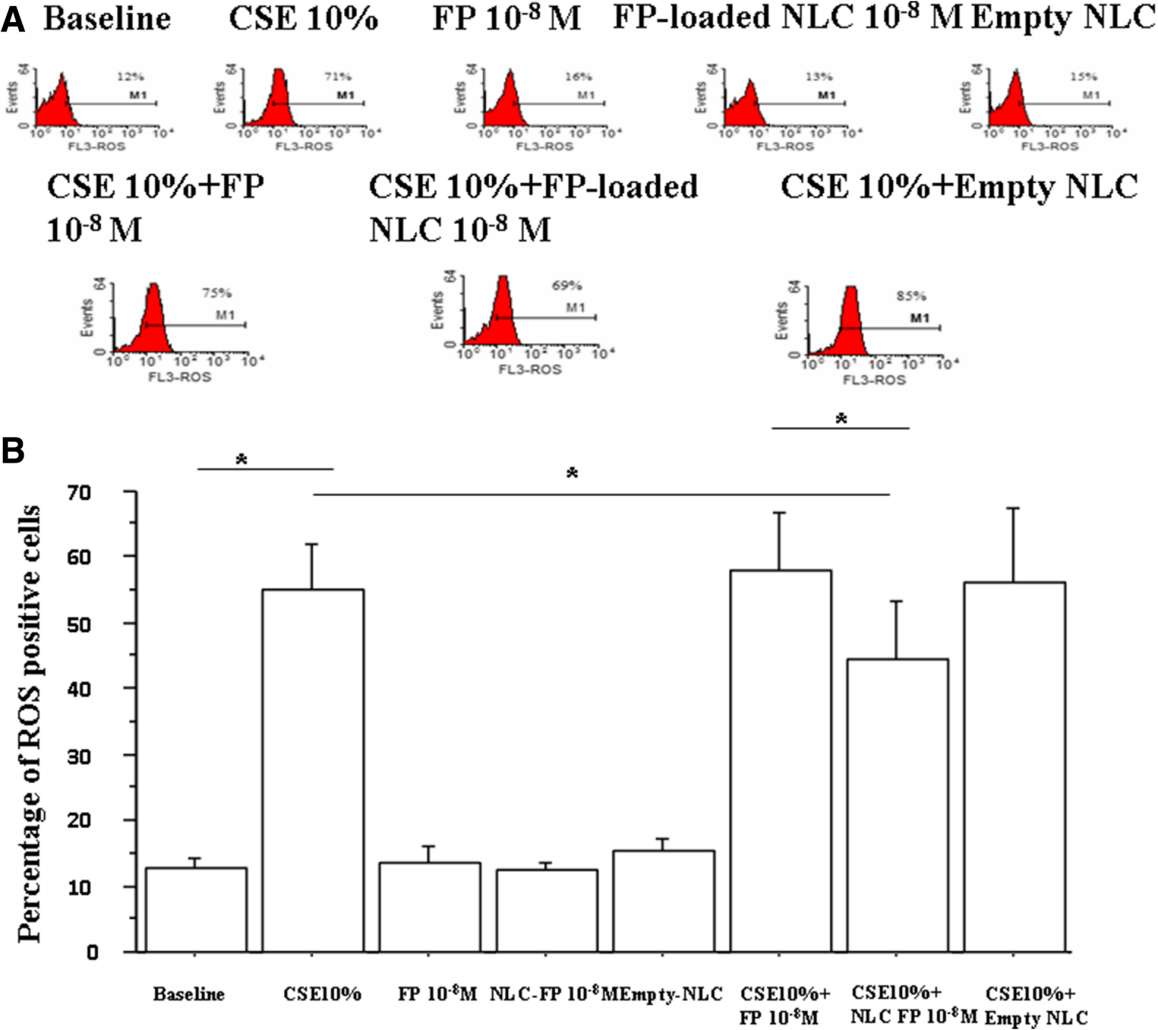
**Effects of FP-loaded NLC on ROS production.** Bronchial epithelial cells (16-HBE) were cultured in the presence and in the absence of CSE (10%), FP, NLC and FP-loaded NLC for 24 hours and then were used for assessing ROS production by flow cytometry (see Materials and methods for details). **(A)** Data are expressed as percentage of ROS positive cells ± SD. *p < 0.05. **(B)** Representative histogram plots are shown.

The increased oxidative stress present in COPD patients is related to the increased burden of inhaled oxidants such as cigarette smoke and to the increase in ROS generated by several inflammatory, immune, and structural airways cells [18]. We initially tested the effect of CSE in ROS production by bronchial epithelial cells. When the cells were exposed to CSE, an increased ROS expression occurred. The presence of free FP tended to increase ROS expression in CSE stimulated cells but this increase was not statistically different. FP-loaded NLC significantly reduced the CSE induced ROS expression and the effect was significantly greater than that exerted by free FP (Figure 5A and B).

**Figure 6:**
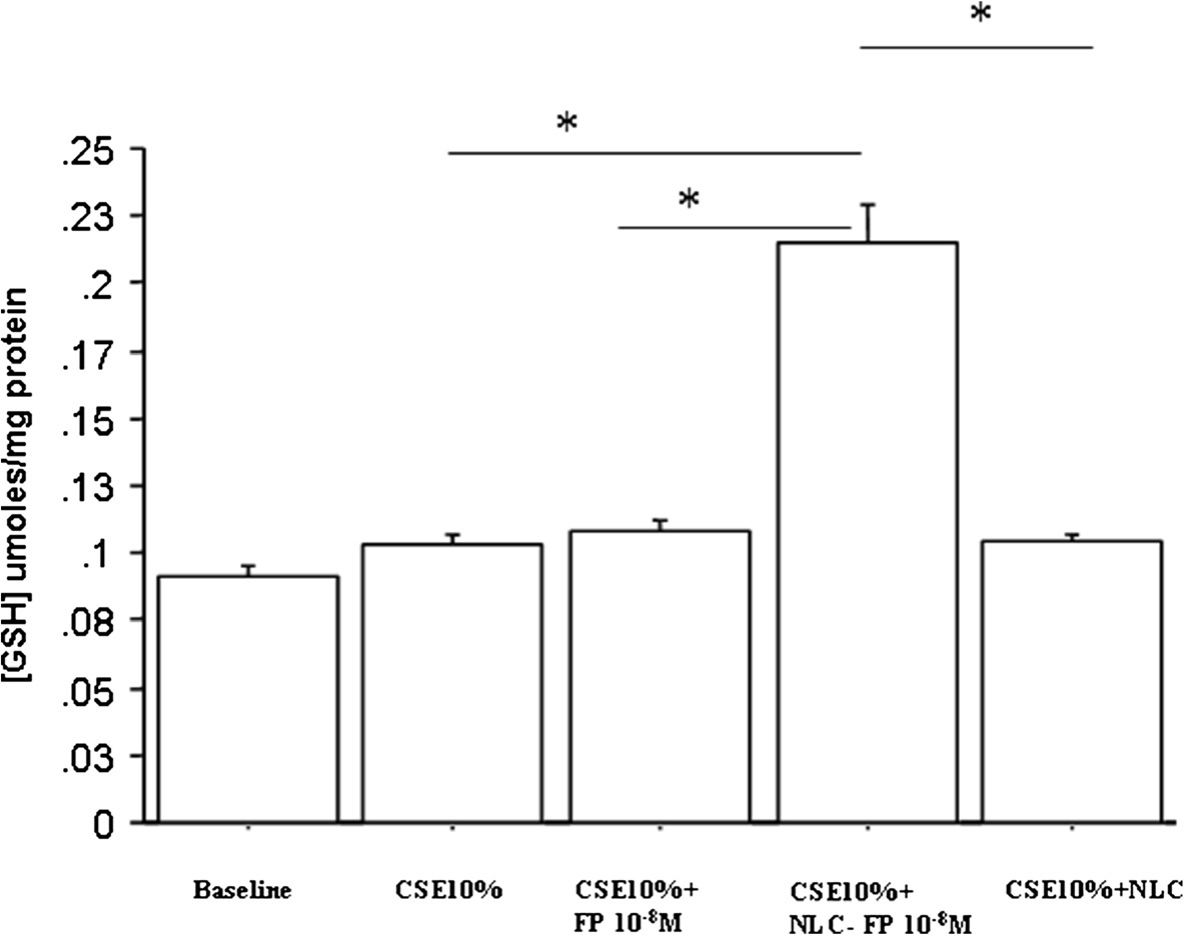
**Effects of FP-loaded NLC on GSH expression.** Bronchial epithelial cells (16-HBE) were cultured in the presence and in the absence of CSE (10%), FP, NLC and FP-loaded NLC for 24 hours and then were used for assessing GSH content (see Materials and methods for details). Data are expressed as GSH μmoles/mg proteins ± SD. *p < 0.05.

Glutathione (GSH) is one of the most important defensive mechanisms against oxidative stress [19,20]. The effects of unloaded FP and FP-loaded NLC as GSH expression in CSE stimulated bronchial epithelial cells were explored. CSE did not significantly increase GSH expression in bronchial epithelial cells. Free FP and empty NLC did not significantly induce GSH expression (data not shown). On the contrary FP-loaded NLC significantly increased GSH expression in CSE stimulated bronchial epithelial cells (Figure 6).

Therefore the increase of GSH suggests a protective effect against oxidative stress into the cells induced by FP when it is administered by NLC.

**Figure 7:**
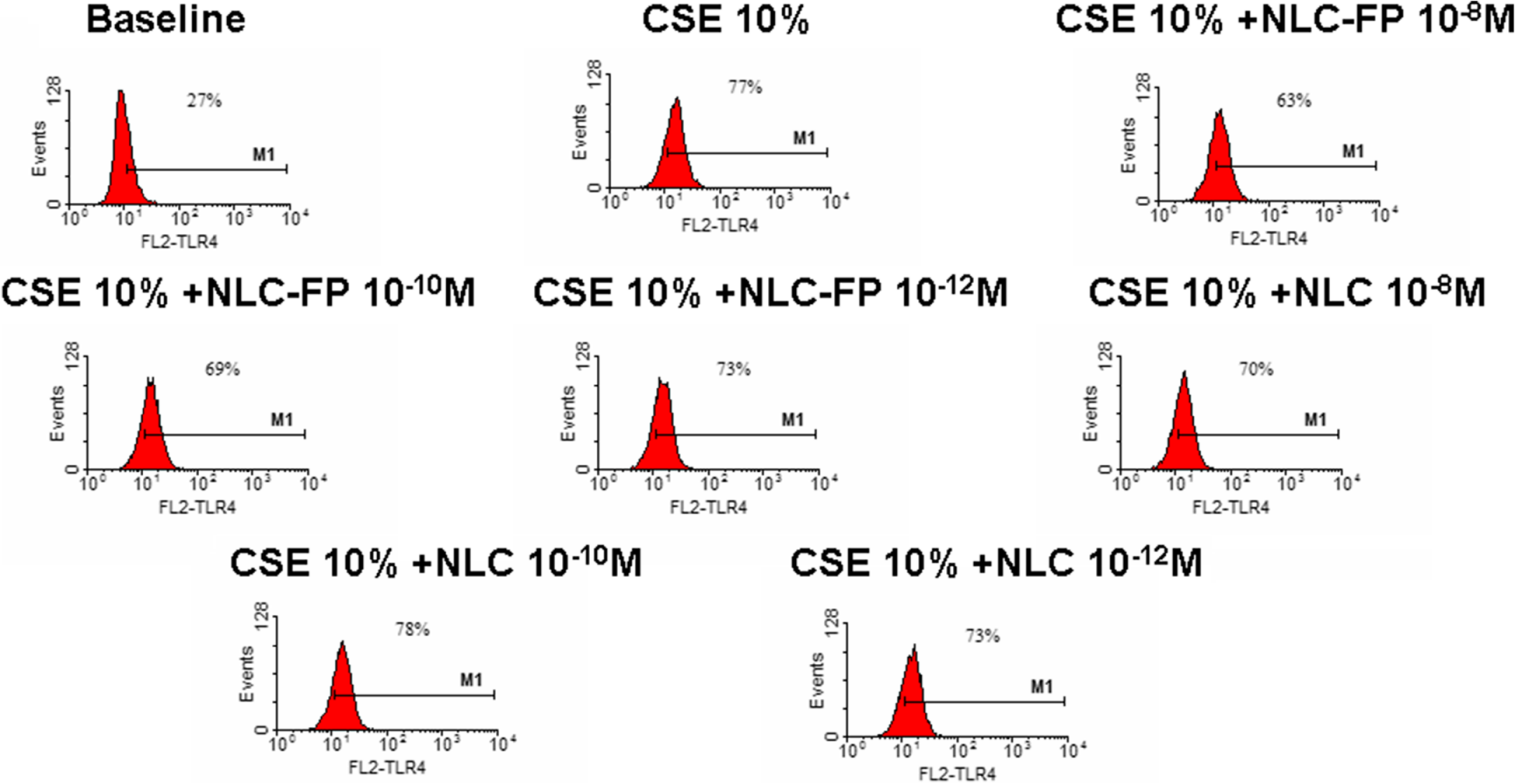
**Dose–response experiments for TLR4 expression.** Bronchial epithelial cells (16-HBE) were cultured in the presence and in the absence of CSE (10%) and FP-loaded NLC at different drug concentrations (10^−8^M, 10^−10^M, 10^−12^M) for 24 hours. Representative histogram plots were shown.

**Figure 8:**
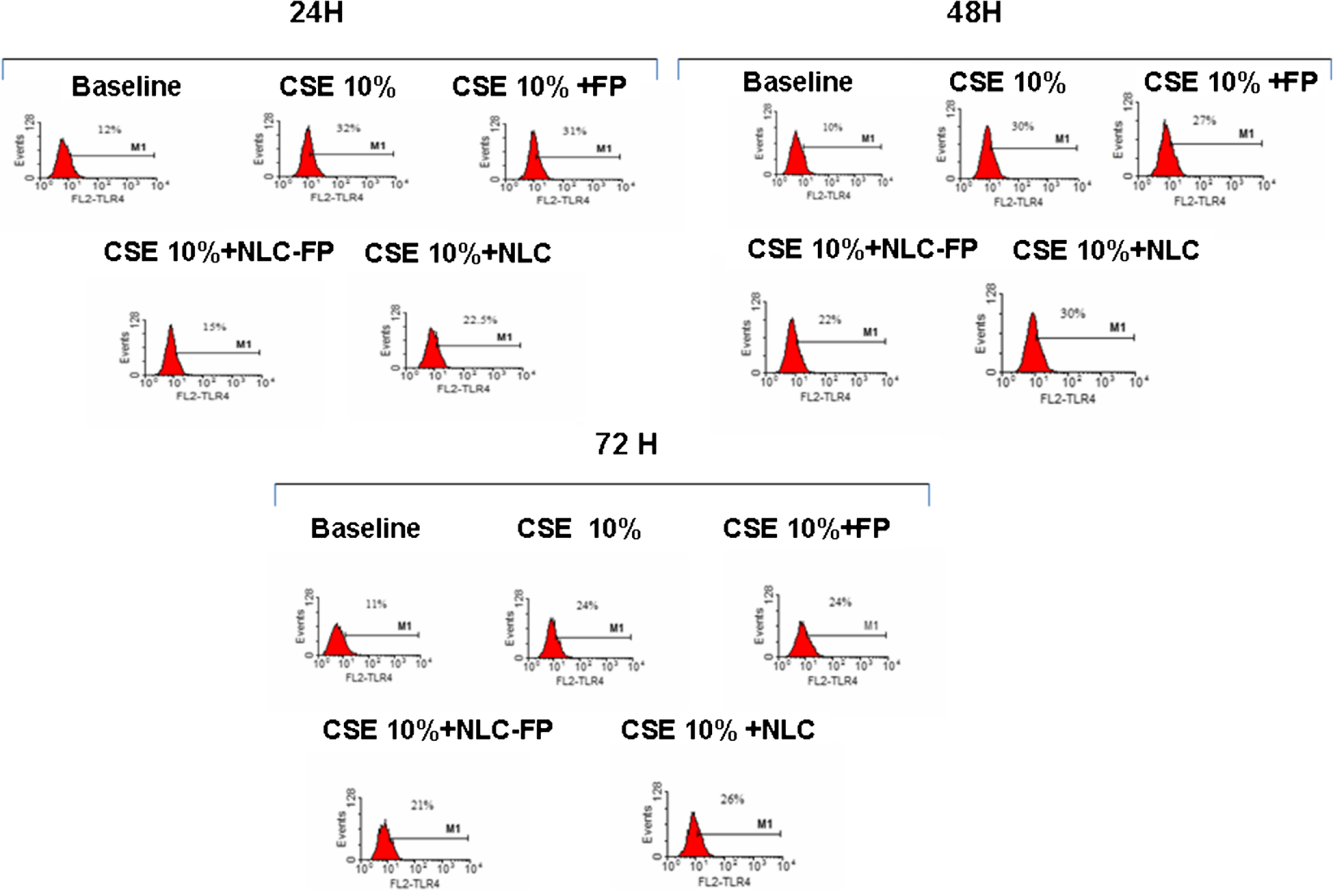
**Time-dependent experiments for TLR4 expression.** Bronchial epithelial cells (16-HBE) were cultured in the presence and in the absence of CSE (10%) and FP-loaded NLC (10^−8^M) for different time points (24, 48 and 72 hours). Representative histogram plots were shown.

**Figure 9:**
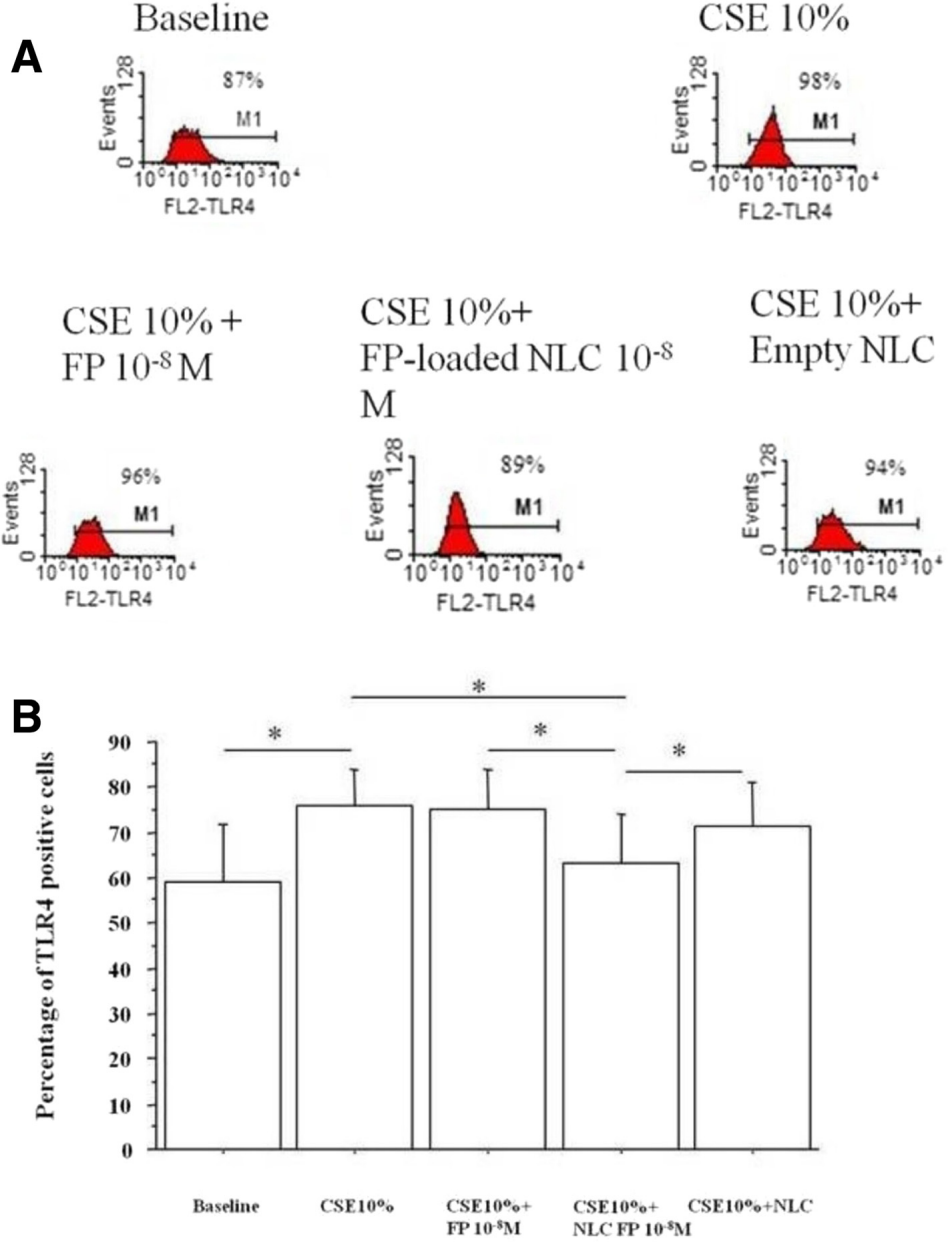
**Effects of FP-loaded NLC on TLR4 expression.** Bronchial epithelial cells (16-HBE) were cultured in the presence and in the absence of CSE (10%), FP, NLC and FP-loaded NLC (10^−8^M) for 24 hours and then were used for assessing TLR4 expression by flow cytometry. **(A)** Representative histogram plots are shown. **(B)** Data are expressed as percentage of TLR4 positive cells ± SD. *p < 0.05.

A key component of the innate immunity and of the innate defence mechanisms against infections is represented by the toll like receptor (TLR4) family. After stimulation of these receptors the cell is triggered to produce inflammatory mediators. Since CSE increased TLR4 expression in bronchial epithelial cells [21,22], the effect of free FP and FP-loaded NLC in CSE induced TLR4 expression was assessed. FP-loaded NLC at 10^−8^M concentration was more effective in reducing TLR4 expression in CSE stimulated cells in comparison to the other two tested concentrations (Figure 7). According to the results of these experiments, 24 hours of incubation was selected as the best time point (Figure 8). Free FP as well as empty NLC did not significantly affect the CSE induced TLR4 expression while FP-loaded NLC significantly reduced the CSE induced TLR4 expression (Figure 9A and B).

**Figure 10:**
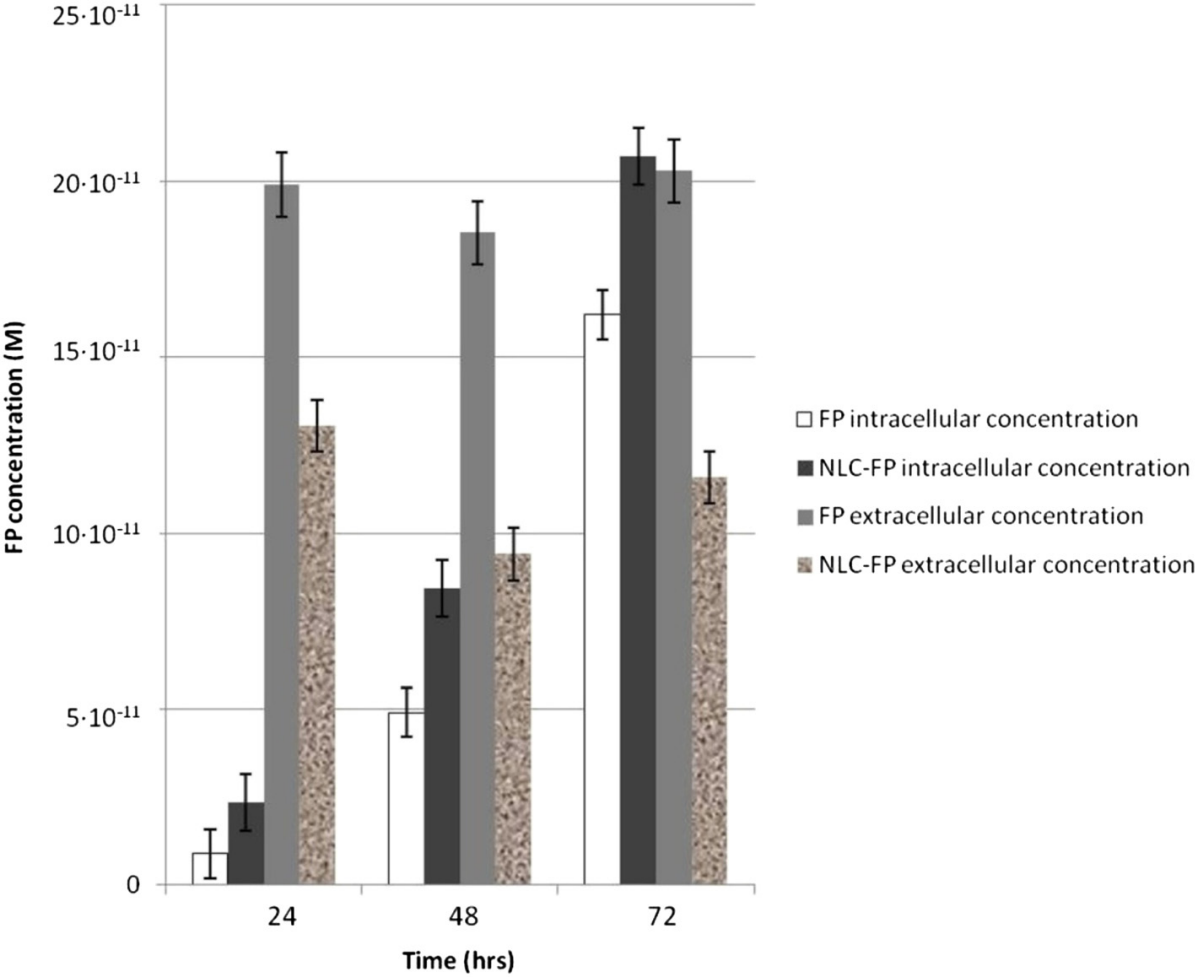
**Intracellular and extracellular concentrations of FP at 24, 48 and 72 hrs.** Data are expressed as Molarity.

To further investigate why the use of FP-loaded NLC was more effective than the free drug in reducing CSE-mediated effects, the intracellular and extracellular contents of FP in 16-HBE cells treated with unloaded FP or with FP-loaded NLC were assessed by UV analysis. The content of FP was higher within the cells treated with FP-loaded NLC than within the cells treated with free FP at all time points. These results further supported the obtained biological data. Figure 10 shows that the intracellular concentrations of FP loaded into the NLC were always higher than those found in cells treated with free FP. Furthermore, the intracellular and extracellular concentrations were lower than the ones used for the experiments because an aliquot of FP was probably degraded by enzymes present in the cells, thus confirming the data reported in the literature [23].

Cigarette smoking is the major cause of chronic obstructive pulmonary disease, which is associated with increased oxidative stress and altered innate and adaptive immunity [24].

Cigarette smoke-mediated oxidative stress other than producing protein denaturation [25], lipid peroxidation, and DNA damage, contributes to reducing corticosteroid activity [26]. In the presence of mucus hypersecretion, a phenomenon frequently present in the airways of COPD patients, lipophilic substances, such as corticosteroids, can be remarkably impeded in reaching their receptors, which are localized within the cytoplasm of bronchial epithelial cells. A modified delivery system that provides constant levels of drug at the prime site of action for a prolonged time can contribute to better control this disease [27]. Lipid nanoparticles such as NLC may increase cell uptake of the drug and may improve drug stability and these events may contribute to increased efficacy of the drug. By varying the composition of lipids, structure and size, they could offer a controlled and prolonged duration of the effect of the encapsulated drugs as well as a regional and cell-specific drug targeting within the airways. This kind of drug carriers presents many advantages [28,29].

On the basis of the well-known capability of the NLC to solubilize adequate amounts of hydrophobic drugs, to pass through the mucus layer associated with bronchial inflammatory diseases escaping from pulmonary phagocytosis due to their bulky hydrophilic outer shell [11], the potential of NLC containing PEG chains as inhalatory delivery systems for FP, was investigated.

The present study describes the preparation of NLC loaded with FP and demonstrates their biocompatibility and their efficacy in controlling oxidative stress and innate immune responses in bronchial epithelial cells exposed to cigarette smoke extracts. The method used for preparing cigarette smoke extracts was previously validated [30,31] and samples of CSE were filtered to remove bacteria and other macromolecules. These large particles in vivo do not reach the deeper airways because they are deposited in the oral cavity or in the upper respiratory tract. Furthermore, CSE 10% was selected because we showed that this concentration was not toxic for the cells and was able to significantly increase both ROS production [17] and TLR4 expression [21] in bronchial epithelial cells. In addition, this concentration as mentioned in a previous study [32] corresponds to exposures associated with smoking equivalent to two packs per day of cigarettes.

Lungs are unique because they have a large epithelial surface area that is at risk for oxidant-mediated attack. The tracheobronchial tree and the alveolar space are exposed to reactive oxidizing species in the form of inhaled airborne pollutants, tobacco smoke, and products of inflammation. The ROS play an integral role in the modulation of several physiological functions but can also be destructive if produced in excessive amounts. Cigarette smoke results in an imbalance between oxidants and antioxidants in favor of oxidants thus promoting increased oxidative stress [33]. Oxidative stress leads to cause oxidative lung damage including apoptosis [34], senescence and inflammation, all of which have been described in the airways of smokers with COPD [23]. Signal transducers and activators of transcription (STAT), nuclear factor-κB, and transcription factor activator protein-1 (AP-1) are activated in epithelial cells and inflammatory cells during oxidative stress [35]. Increased oxidative stress leads to reduced histone deacetylase (HDAC) activity contributing to the low response to corticosteroids [36]. Upon the exposure of cigarette smoke extracts in airway epithelial cells, the ubiquitin proteasome system is unable to cope with severely damaged proteins that accumulate in the cell in the form of insoluble polyubiquitinated aggregates [37]. In the present study we confirm that CSE increase ROS and here we provide data supporting the efficacy of FP when it is entrapped into NLC containing PEG reducing TLR4 expression any more than compared to NLC without PEG (see below) and thus suggesting an important role of the PEG nanoparticles in reducing the effects of CSE in the modulation of innate immunity responses. Although the majority of COPD cases can be directly related to smoking, only a quarter of smokers actually develop the disease. A potential reason for the disparity between smoking and COPD may involve an individual’s ability to mount a protective adaptive response to cigarette smoke. The GSH system belongs to enzymatic anti-oxidant systems. It is highly concentrated in the lung epithelial lining fluid and protects against many inhaled oxidants. The exposure to cigarette smoke in airway epithelial cells leads to the exhaustion of the pool of reduced GSH thus promoting a lack of antioxidant protection [38]. Under our experimental conditions in vitro CSE do not increase GSH levels as well as no effect on GSH levels were recorded by using free FP. On the contrary we provide data supporting the efficacy of FP-loaded NLC in increasing GSH levels in the presence of CSE.

Furthermore, the surface of the airway epithelium represents a battleground in which the host intercepts signals from pathogens and external insults and activates epithelial defences mainly represented by the innate host immune system. Innate immunity relies on pattern recognition receptors that recognize molecular structures common to many micro-organisms, such as lipopolysaccharides (LPS), and endogenous ligands such as heat shock proteins. TLR4 is a transmembrane protein that participates in the recognition of LPS and plays a crucial role in the activation of innate host immune system. LPS via TLR4 activation increases inflammatory responses and stimulates MUC5AC expression thus contributing to airway mucus hypersecretion [39]. TLR4 expression is increased in the bronchial epithelium of smokers [40] and cigarette smoke increases TLR4 expression in bronchial epithelial cells [21] and, via activation of the TLR4 signaling cascade, mediates MMP-1 expression [41] and increases IL-8 release [42]. All together these findings suggest that the over-expression and over-activation of TLR4 can contribute to many phenomena associated to COPD pathogenesis. In the present study we confirm that CSE increase TLR4 expression and demonstrate that FP-loaded NLC, but not unloaded FP, significantly reduce the effects of CSE in increasing TLR4 expression. Moreover, when FP is entrapped into the pegylated NLC is more effective than FP entrapped into the un-pegylated NLC (data not shown).

## Conclusions

In the present study NLC based on a pegylated lipid have been prepared and tested as carrier of FP. Either empty and drug-loaded NLC showed negative ζ potential values and a mean size in the nanometer scale with low PDI values, which indicated a good dimensional homogeneity of particles such as make them suitable for inhalatory administration.

Excellent stability was also showed by this system during storing in the dried form at 4°C, being unchanged their size, PDI and ζ potential.

The release study showed that the investigated carrier had a great stability, being able to retain about 80% of initially entrapped corticosteroid even after 72 h. This result is in agreement with the hypothesis that this system, able to keep inside the drug, at the contact with airways mucosae, could improve drug cell uptake because FP-loaded NLC could enter the cells by endocytosis.

On the other hand a greater amount of FP was found into bronchial epithelial cells treated with FP-loaded NLC in comparison with that treated with free FP.

*In vitro* studies on 16-HBE cells revealed that neither unloaded FP nor FP-loaded NLC induced relevant numbers of necrotic or of apoptotic cells. In 16-HBE cells exposed to CSE, FP-loaded NLC were able to control oxidative stress increasing oxidant/anti-oxidant balance in favour of anti-oxidant responses and to limit innate immune responses, and were similar or superior to unloaded FP in these effects. These observations suggest the use of this system for the FP administration in inhalation therapies because of the ability of NLC to solubilize an adequate amount of the drug and to penetrate into the airway epithelial cells. These findings suggest a potential role of these nanocarriers in the therapy of chronic obstructive pulmonary diseases such as COPD.

## Experimental

### Materials and methods

Fluticasone propionate, sodium taurocholate, and acetonitrile for HPLC were purchased from Sigma Aldrich (Milan, Italy). Dichlorometane for HPLC was obtained from Merck (Germany). Compritol HD5 ATO (behenoyl polyoxyl-8 glycerides) was a gift sample from Gattefossè (France). Epikuron 200 (soybean lecithin) was a gift sample from Lucas Meyer Company (Germany). HPLC (UFLC-Prominence system, Shimadzu Instrument, Japan) was equipped with two pumps LC-20 AD, an UV-visible detector SPD-20 AV, an autosample SIL-20A HT and a column Gemini® C_18_ Phenomenex (250 mm, 5 μm particle size, 110 Å pores size).

### Preparation of NLC

Pegylated NLC, empty or FP-loaded were prepared by the precipitation method [8-10]. Briefly, Compritol HD5 ATO (180 mg) was heated at 5–10°C above its melting point (m.p. 60°-67°C). For obtaining drug-loaded NLC, FP (10 mg; m.p. 272°-273°C) was added, under mechanical stirring, to the melted lipid phase. An ethanolic solution (2 ml) of Epikuron 200 (78.5 mg) was then added to the melted lipid phase containing FP and the resulting organic dispersion was dispersed into bidistilled water (100 ml) containing sodium taurocholate (177.4 mg) at 2-3°C and stirred by using an Ultraturrax T125 (IKA Labortechnik, Germany) at 13,500 rpm for 10 minutes. Finally, the colloidal aqueous dispersion of NLC was purified by exhaustive dialysis (dialysis tube with 12,000/14,000 Dalton cut-off (Spectra/Por®, USA) and freeze-dried. NLC samples (m.p. about 70°C) were stored at 4 ± 1°C for successive characterization.

### Scanning Electron Microscopy (SEM) analysis

For morphological studies, freeze-dried samples were observed by using an ESEM FEI Quanta 200F scanning electron microscope. Samples were dusted on a double-sided adhesive tape, previously applied on a stainless steel stub. All samples were then sputter-coated with gold prior to microscopy examination.

### Particle size analysis

The hydrodynamic diameter (*z*-average) and the width of distribution (polydispersity index, PDI) of the nanosuspensions were investigated by Photon Correlation Spectroscopy (PCS) by using a Zetasizer Nano ZS (Malvern Instrument Ltd, UK). The nanoparticles were diluted until the appropriate concentration and then the measurements performed at a temperature of 25°C, at a fixed angle of 173° (NIBS = non-invasive backscattering detection) in respect to the incident beam. Bidistilled water, isotonic aqueous solution (NaCl 0.9% w/w), and phosphate buffered saline solution (PBS) at pH 7.4 as suspending media were used. When the measurement was carried out in NaCl 0.9 wt%, the instrument setting conditions were: μ = 0.902, RI = 1.331; in PBS at pH 7.4, the setting conditions were: μ = 0.980, RI = 1.334. Results of light scattering experiments are given as the average values obtained using samples from three different batches. Each sample was measured in triplicate.

### ζ potential measurements

The surface charge or ζ potential is considered as one of the benchmark of stability of a colloidal system. It indicates the degree of repulsion between similarly charged particles into a dispersion. For the nanoparticles, a high value of ζ potential will confer stability and the nanosuspensions will resist aggregation phenomena. When the ζ potential is low, attraction exceeds repulsion and the dispersions will flocculate.

The analysis was performed at a temperature of 25 ± 1°C using appropriately diluted samples in the same media used for size measurements. Instrument setting conditions were equal to those described above for size measurements.

Results of these experiments are given as the average values obtained using samples from three different batches. Each sample was measured in triplicate.

### HPLC analysis

An adequate HPLC method was developed to reveal FP and to study its stability in PBS at pH 7.4, as well as Loading Capacity (LC%) and drug release profiles from drug-loaded NLC. The HPLC analysis was performed at room temperature by using the instrument described above. A column Gemini® C_18_ Phenomenex (above described) was used as stationary phase and a mixture of CH_3_CN/H_2_O 80/20 (v/v), with a flow rate of 0.8 ml/min, was used as mobile phase with an isocratic method. The drug peak was measured at wavelength of 239 nm and quantitatively determined by comparison with a standard curve obtained using FP organic solutions in a mixture of CH_2_Cl_2_:CH_3_CN 3:2 (v/v) at known concentrations (t_r_ =7.03 min). The straight-line equation was: y = 4 • 10^5^ × and the linear regression value was: r^2^ = 0.9993. The linearity of the method was studied in the range 0.30-1.20 μg/ml.

### Drug loading and entrapment efficiency determination

Loading capacity (LC%) was determined by solubilizing the nanoparticles into an organic mixture (CH_2_Cl_2_:CH_3_CN 3:2 (v/v)), filtered with 0.45 μm PTFE syringe filters (Puradisc Whatman) and analyzed by the HPLC method described above. Drug loading capacity (DL%) was calculated as drug analyzed in the nanoparticles versus the total amount of the drug and the lipid added during preparation, according to the following equation, where *W*_*drug*_ is the amount drug found inside nanoparticles and *W*_*NPS*_ is the weight of drug-loaded nanoparticle:

$$ DL\ \%=\frac{W_{drug}}{W_{NPs}}\times 100 $$

Results are given as the average values obtained using samples from three different batches and were expressed as the percentage of the FP amount contained in 100 mg of dried material (LC%). Moreover, entrapment efficiency (*EE%*) was determined using the HPLC method above described on purified NLC following their disruption with a mixture of CH_2_Cl_2_ and CH_3_CN (3:2 v/v). The encapsulated amount of FP was expressed dividing the found amount of FP and the total amount used to prepare the nanoparticles. The following equation was used to calculate the *EE%*, where *W*_*f*_ is the amount drug found and *W*_*i*_ is the initial amount of drug for the preparation:

$$ EE\ \%=\frac{W_f}{W_i}\times 100 $$

### Stability studies in PBS/ethanol

In order to obtain a release profile of FP from NLC under sink conditions, a mixture of PBS at pH 7.4 and ethanol 80:20 (v/v) was used. The term “sink conditions” refers to release conditions in which the volume of the buffer used is sufficient to dissolve all drug present into NLC. Such conditions are used to assure that the amount of drug released is not limited by the degree of solubility in the buffer or solvent used. In particular, lyophilized FP-loaded nanoparticles (5 mg) were suspended into the mixture release medium above described (5 ml) and transferred inside of a Spectra/Por® dialysis membrane that was immersed into the same pre-heated medium (25 ml) and incubated at 37 ± 0.1°C, under continuous stirring, in a Benchtop Incubator Orbital Shaker model 420 (Thermo-Scientific Instruments, CA).

At scheduled time, solution aliquots were taken out from the outside of the dialysis membrane and replaced with equal volumes of the fresh PBS/ethanol mixture. In order to determine the released FP amount, the drawn samples were filtered by 0.2 μm cellulose syringe filters (Millipore) and analyzed by HPLC, following the method above described. Profile releases were determined by comparing the amount of released drug as a function of incubation time with the total amount of drug loaded into the nanoparticles.

Moreover, in order to determine the amount of FP entrapped into residual NLC samples, the PBS suspension containing FP-loaded NLC was freeze-dried (FreeZone®Freeze Dry System, Labconco Corporation, Missouri, USA). Successively, an organic mixture (CH_2_Cl_2_:CH_3_CN 3:2 (v/v)), was added to lyophilized product, which was filtered through 0.2 μm (PTFE membrane) filters and analysed by HPLC, as reported above.

Finally, in order to determine the diffusion behaviour of the unloaded drug a control experiment was also performed. At this purpose, an appropriate amount of FP (equal to whom of FP-loaded nanoparticles) was dispersed in the mixture release medium (5 ml), placed inside a dialysis tube (MWCO 12,000-14,000 Da) and immersed into the same medium (25 ml). The amount of FP was detected by HPLC, as reported above.

### Storage and colloidal stability evaluation

Both lyophilised empty and FP-loaded NLC were stored at 4°C for 4 and 10 months in the dark. The stability test was carried only at 4°C and not at room temperature because the NLC are prepared with lipids that must be stored at a temperature not exceeding 10°C so as reported in the data sheet of the supplier of Gattefosse Compritol HD5 ATO. After this period of storage, samples were dispersed in bidistilled water and characterized in terms of mean size, PDI, ζ potential and drug stability.

#### Preparation of cigarette smoke extracts (CSE)

Commercial cigarettes (Marlboro) were used in this study. Cigarette smoke solution was prepared as described previously [21]. Each cigarette was smoked for 5 min and two cigarettes were used per 20 ml of PBS to generate a CSE-PBS solution. The CSE solution was filtered through a 0.22 μm-pore filter to remove bacteria and large particles as previously described and standardised [30,31]. The smoke solution was then adjusted to pH 7.4 and used within 30 minutes of preparation. This solution was considered to be 100% CSE and diluted to obtain the desired concentration in each experiments. The concentration of CSE was calculated spectrophotometrically measuring the optical density (OD) as previously described at the wavelength of 320 nm [21]. The presence of contaminating LPS on undiluted CSE was assessed by a commercially available kit (Cambrex Corporation, East Rutherfort, New Jersey, USA) and was below the detection limit of 0.1 EU/ml.

### Stimulation of bronchial epithelial cell lines

The SV40 large T antigen-transformed 16-HBE cell line (16-HBE) was used for these studies [21]. 16-HBE is a cell line that retains the differentiated morphology and function of normal airway epithelial cells. 16-HBE was maintained in Eagle’s minimum essential medium (MEM) supplemented with 10% heat-inactivated (56°C, 30 min) fetal bovine serum (FBS), 1% MEM (non-essential amino acids, Euroclone), 2 mM L-glutamine and gentamicin 250 μg/ml. Cell cultures were maintained in a humidified atmosphere of 5% CO2 in air at 37°C. 16HBE were plated in 12-well plates. 70.000 cells in 1ml MEM 10% FBS were seeded for each well. At confluence 16-HBE cells were treated in 1 ml MEM 1% FBS in presence of CSE (10%) and with or without FP (10^−8^M), empty-NLC or FP-loaded NLC (10^−8^M) for 24 hrs. 1% FBS was used during cell stimulation to limit the basal activation of the cells due to serum proteins. Preliminary experiments aimed to identify the best time point (24, 48 and 72 hrs) as well as the best drug concentration (FP-loaded NLC 10^−8^M, 10^−9^M, 10^−10^M) were performed. At the end of stimulation, cells were collected for further evaluations.

### Cell apoptosis by annexin V binding method

Cell apoptosis in the presence of free FP (10^−8^M), empty NLC and FP-loaded NLC (10^−8^M) was evaluated by staining with annexin V-fluorescein isothiocyanate and propidium iodide (PI) using a commercial kit (Bender MedSystem, Vienna, Austria) following the manufacturer's directions**.** Cells were analyzed using a FACS Calibur (Becton Dickinson, Mountain View, CA) analyzer equipped with an Argon ion Laser (Innova 70 Coherent) and Consort 32 computer support.

### Analysis of intracellular reactive oxygen species (ROS)

Intracellular ROS were measured by the conversion of the non-fluorescent dichlorodihydrofluorescein diacetate (DCFH-DA; Sigma Aldrich, Milan, Italy) in a highly fluorescent compound, dichlorofluorescein (DCF), by monitoring the cellular esterase activity in the presence of peroxides. The ROS generation was assessed by uptake of 1 μM DCFH-DA, incubation for 10 min at room temperature in the dark, followed by flow cytometric analysis.

### Measurement of cellular glutathione (GSH) content

Intracellular total GSH content was assessed in cell extracts as previously reported [43]. Briefly, cell extracts were prepared in 0.1 M potassium phosphate extraction buffer containing 0.6% (w/v) sulfosalicylic acid, 0.1% (v/v) Triton X-100, 5 mM EDTA. After harvesting and resuspension in extraction buffer, cells were sonicated in ice-cold water and underwent two cycles of freezing and thawing. Supernatants/extracts were collected by centrifugation and used for the following colorimetric assay: 10 μl of extracts were incubated in presence of 60 μl 0.6 mg/ml 5,5′-Dithiobis(2-nitrobenzoic acid) (DTNB) and 60 μl of 250 U/ml glutathione reductase for 30 seconds at room temperature; 50 μl of 0.6 mg/ml β-NADPH were added and formation of 2-nitro-5-thiobenzoic acid was immediately evaluated by measuring the absorbance at 412 nm in a microplate reader. Concentration of GSH in cell extracts was calculated using a standard curve, normalized by the total protein content and expressed as nmol/mg protein.

### Expression of TLR4 in 16-HBE

The total TLR4 protein expression (inside the cells and on their surface) was assessed in permeabilized cells. For cell permeabilization, a commercial fix-perm cell permeabilization kit (Caltag Laboratories, Burlingame, CA, USA) was used. Cells were incubated in the dark (30 min, 4°C) with PE anti-human TLR4 monoclonal mouse antibody (eBioscence San Diego CA) and then evaluated by flow-cytometry (FACS Calibur).

Negative controls were performed using mouse immunoglobulins negative control (Dako). Data are expressed as percentage of positive cells.

### Intracellular and extracellular concentrations of FP and NLC-FP

Cell cultures were maintained in a humidified atmosphere of 5% CO_2_ in air at 37 ± 1°C. Cell lines were cultured in the presence and in the absence of free FP (10^−8^M) and FP-loaded NLC (10^−8^M) for 24, 48 and 72 hrs. At the end of stimulation, cells and supernatants were collected for assessing the intracellular and extracellular content of FP by UV analysis. In particular, the supernatants were sucked from the wells and then collected by centrifugation at 1300 rmp for 10 min. Cells were detached from the wells by tripsin, washed with PBS and stored as dry pellet at −20°C. After several cycles of freezing and thawing, the cells as well as the previously recovered culture supernatants, were used for testing their FP content. FP was extracted both from the cells from the supernatants with an organic solution (4 ml) of CH_2_Cl_2_:CH_3_CN (3:2 v/v), filtered with 0.45 μm PTFE syringe filters (Puradisc Whatman) and the absorbance was determined by ultraviolet–visible (UV–vis) Spectrophotometer (UV-1800 Shimadzu, Kyoto, Japan) at 239 nm.

### Statistics

Data are expressed as mean counts ± standard deviation. Comparison between different experimental conditions was evaluated by paired *t* test. P < 0.05 was accepted as statistically significant.

## Abbreviations

AP-1: Activator protein-1
COPD: Chronic obstructive pulmonary disease
CSE: Cigarette smoke extracts
DCF: Dichlorofluorescein
DCFH-DA: Dichlorodihydrofluorescein diacetate
DL: Drug loading capacity
DTNB: 5,5′-Dithiobis(2-nitrobenzoic acid)
EDTA: Ethylenedyaminetetraacetic acid
EE: Entrapment efficiency
FP: Fluticasone propionate
FBS: Fetal bovine serum
GSH: Glutathione
16-HBE: Human bronchial epithelial cell line
HDAC: Histone deacetylase
HPLC: High pressure liquid chromatography
ICS: Inhaled corticosteroids
LC: Loading capacity
LPS: Lipopolysaccharides
MEM: Eagle’s minimum essential medium (MEM)
NADPH: Nicotinamide adenine dinucleotide phosphate
NLC: Nanostructured lipid carriers
OD: Optical density
PBS: Phosphate buffered saline solution
PCD: Photon correlation spectroscopy
PDI: Polydispersity index
PI: Propidium iodide
ROS: Reactive oxygen species
SEM: Scanning electron microscopy
STAT: Signal transducers and activators of transcription
TLR4: Toll-like receptor 4
